# A Review of Pathophysiology and Therapeutic Strategies Targeting TGF-β in Graves’ Ophthalmopathy

**DOI:** 10.3390/cells13171493

**Published:** 2024-09-05

**Authors:** Hsin-Ho Chang, Shi-Bei Wu, Chieh-Chih Tsai

**Affiliations:** 1 Department of Ophthalmology, Taipei Veterans General Hospital, Taipei 112, Taiwan; 2 Office of Business Development, Technology Commercialization Center, Taipei Medical University, Taipei 110, Taiwan; 3 Department of Ophthalmology, School of Medicine, National Yang Ming Chiao Tung University, Taipei 112, Taiwan

**Keywords:** Graves’ ophthalmopathy (GO), transforming growth factor-β (TGF-β), orbital fibrosis, extracellular matrix (ECM), orbital fibroblast

## Abstract

TGF-β plays a pivotal role in the pathogenesis of GO by promoting orbital tissue remodeling and fibrosis. This process involves the stimulation of orbital fibroblasts, leading to myofibroblast differentiation, increased production of inflammatory mediators, and hyaluronan accumulation. Studies have elucidated TGF-β’s role in driving fibrosis and scarring processes through both canonical and non-canonical pathways, particularly resulting in the activation of orbital myofibroblasts and the excessive accumulation of extracellular matrix. Additionally, recent in vitro and in vivo studies have been summarized, highlighting the therapeutic potential of targeting TGF-β signaling pathways, which may offer promising treatment interventions for GO. This review aims to consolidate the current understanding of the multifaceted role of TGF-β in the molecular and cellular pathophysiology in Graves’ ophthalmopathy (GO) by exploring its contributions to fibrosis, inflammation, and immune dysregulation. Additionally, the review investigates the therapeutic potential of inhibiting TGF-β signaling pathways as a strategy for treating GO.

## 1. Introduction

Graves’ ophthalmopathy (GO), an extrathyroidal manifestation of Graves’ disease, characterized by soft tissue inflammation, aberrant tissue remodeling, and fibrosis, represents the most frequent extrathyroidal manifestation of Graves’ disease [1]. Common clinical symptoms of GO include proptosis, eyelid retraction, lagophthalmos, dry eye, peri-orbital swelling, and diplopia. In more severe cases, although less common, orbital deformity and compressive optic neuropathy may also occur. These manifestations may lead to cosmetic concerns and are potentially vision-threatening, significantly impacting both functional abilities and quality of life [2]. 

The molecular and cellular mechanisms of abnormal orbital tissue remodeling in GO involve the activation of orbital fibroblasts, leading to orbital myofibroblast differentiation, activation of T helper cells including Th1, Th2, and Th17 cells, increased secretion of inflammatory cytokines such as IFN-γ, TNF-α, interleukins, and prostaglandin E2, as well as hyaluronan production and deposition in the extracellular matrix (ECM), and adipogenesis [3]. These processes ultimately cause fibrosis, thickening of both extraocular muscles and adipose tissue with inflammatory cell infiltration, and volume expansion of orbital tissue.

Transforming growth factor-β (TGF-β) has been implicated in various systemic fibrotic diseases and autoimmune disorders. Numerous studies have demonstrated the critical role of TGF-β in fibrosis and orbital remodeling in GO, primarily through activating myofibroblasts, accumulating excessive extracellular matrix (ECM), and promoting inflammation [4,5]. Therefore, targeting TGF-β signaling pathways holds promise for the development of novel therapeutic interventions for GO. Herein, this review provides an overview of the current understanding of TGF-β’s involvement in GO, emphasizing its role in the pathophysiology of fibrosis and immune inflammatory responses, along with possible therapeutic targets for GO. 

## 2. TGF-β Superfamily

The cytokines belonging to the TGF-β superfamily are dimeric proteins known for their conserved structures and multifunctional roles. TGF-βs, consisting of three isoforms (TGF-β1, TGF-β2, and TGF-β3) in mammals, regulate diverse signaling pathways in proliferation, differentiation, and the maintenance of homeostasis [6]. The three isoforms share similar biologically active regions and bind to the same type I and type II TGF-β receptor complex, while TGF-β1 is the most widely expressed isoform [7]. Additionally, previous in vitro studies have shown that TGF-β1, but not TGF-β2, induces the fibrotic process in orbital fibroblasts, accompanied by increased levels of CTGF, fibronectin, and α-SMA in patients with GO [8]. Primarily, TGF-βs promote the synthesis of extracellular matrix proteins, leading to fibrosis in tissues, while also exhibiting growth-inhibitory effects on various cell types and triggering the apoptosis of epithelial cells. Beyond TGF-βs, the TGF-β superfamily encompasses activins, inhibins, nodal, myostatin, bone morphogenetic proteins (BMPs), growth/differentiation factors (GDFs), and anti-Müllerian hormone (AMH) [9]. 

## 3. TGF-β Signaling in Fibrosis and Inflammation

**Figure 1:**
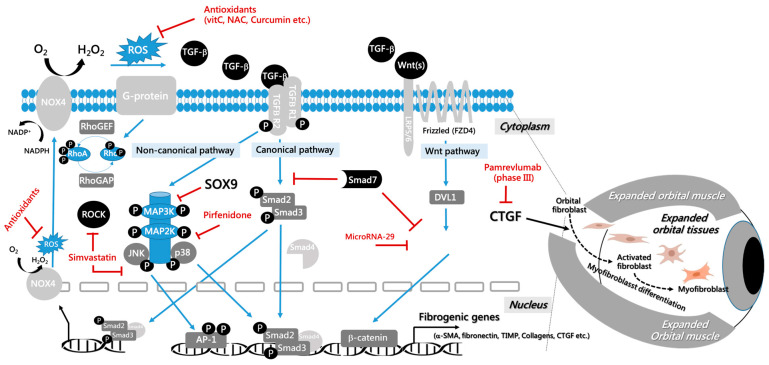
Molecular and cellular mechanisms of TGF-β signaling pathway in GO. TGF-β exerts its multifaceted effects on fibrosis and inflammation through both canonical and non-canonical signaling pathways. It binds to TGFR2, subsequently activating TGFR1. In the canonical pathway, TGFR1 phosphorylates Smad2 and Smad3, enabling their complex formation with Smad4. This complex then translocates to the nucleus, initiating transcription of fibrogenic genes. In non-canonical signaling pathways, TGF-β promotes fibrosis and ECM deposition by activating mitogen-activated protein kinase (MAPK) pathways, phosphoinositide 3-kinase (PI3K) pathways, RhoA-GTPase pathways, Wnt/β-catenin pathways, and NOX4 pathways. In the MAPK pathways, phosphorylated p38/JNK also interacts with the canonical pathway by activating Smad2 and Smad3, c-Jun, and AP-1. Additionally, in the Wnt/β-catenin pathway, Wnt ligands bind to the FZD and LRP5/6 receptors, leading to increased levels of β-catenin. β-catenin then translocates to the nucleus to induce target gene expression. The blue arrow indicates an activating effect, while the red T-bar signifies an inhibitory effect.

TGF-β plays a pivotal role in regulating fibrosis, inflammation, and immune responses [1]. Dysregulated TGF-β expression and signaling are linked to various fibrotic diseases, including renal fibrosis in chronic kidney disease, liver fibrosis in cirrhosis, and orbital fibrosis in GO [4,10]. TGF-β promotes the differentiation of fibroblasts into myofibroblasts and the accumulation of ECM proteins by activating both canonical and non-canonical signaling pathways. This process is essential for tissue remodeling and repair, further underscoring the multifaceted role of TGF-β in orchestrating fibrotic responses. The pathogenesis of the molecular and cellular mechanisms of the TGF-β signaling pathway in GO is summarized in Figure 1.

Overall, TGF-β activation leads to the overexpression of downstream fibrogenic genes and increases in fibrotic markers such as αSMA, CTGF, fibronectin, and collagen. This results in myofibroblast differentiation and excessive ECM production, ultimately causing orbital volume expansion and remodeling in GO.

## 4. TGF-β and Myofibroblast Transdifferentiation in GO

Orbital fibroblasts participate in the fibrosis and autoimmune processes in GO. Orbital fibroblasts, as well as macrophages and adipocytes, produce inflammatory mediators such as TGF-β, interleukin-1, and interleukin-6 upon activation by CD4+ T cells via the ligation of CD40-CD154 [2]. TGF-β induces the transdifferentiation of Thy-1(CD90)-positive orbital fibroblasts into activated myofibroblasts, which are characterized by increased expression of alpha-smooth muscle actin (αSMA) and cytoplasmic actin filaments. α-SMA serves as a marker of myofibroblast differentiation, and its upregulation by TGF-β signaling is a key step in transforming fibroblasts into myofibroblasts. This process leads to increased contractile properties and fibrosis [11].

TGF-β also stimulates the proliferation of orbital fibroblasts, which are derived from retrobulbar soft tissue and extraocular muscles, along with cytokine secretion, including insulin-like growth factor-1 (IGF-1), interleukin-1 alpha (IL-1α), interleukin-4 (IL-4), and platelet-derived growth factor (PDGF). In addition, together with interferon gamma and IL-1α secreted by activated T cells and macrophages, TGF-β also induces the orbital fibroblasts to produce glycosaminoglycans, with predominantly hyaluronan [12,13].

The relationship between TGF-β and IGF-1 is intricate. IGF-1, recognized as an autoantigen in GO, contributes to the disease’s pathogenesis by activating downstream signaling pathways, including MAPK and NF-κB, which drive orbital fibroblast proliferation and inflammation via TSH-stimulated proinflammatory cytokines such as IL-6, IL-8, and TNF-α. Recent evidence from a randomized controlled trial demonstrated that teprotumumab, a monoclonal antibody targeting the IGF-1 receptor (IGF-1R), significantly reduces proptosis in patients with active, moderate-to-severe GO [14]. Moreover, while TGF-β is primarily associated with inhibitory effects on epithelial cell proliferation, it can also modulate IGF-1 activity, as observed in contexts such as neoplastic progression and potentially in GO. The interplay between TGF-β and IGF-1 may synergistically exacerbate GO activity and severity, particularly under conditions of oxidative stress [15].

Overall, TGF-β signaling activates orbital fibroblasts and their differentiation into myofibroblasts, resulting in the formation of fibrotic lesions and tissue scarring, which impair physiological function.

## 5. TGF-β and Excessive ECM Accumulation in GO

In GO, the pathological expansion of orbital volume is significantly influenced by the activation of orbital fibroblasts, which are pivotal in mediating the excessive accumulation of ECM and enhancing the infiltration of inflammatory cells [16]. Central to this process is TGF-β signaling, which not only promotes orbital transformations that are highly proficient in producing ECM proteins—but also suppresses the activity of ECM-degradation enzymes. This dual action fosters an environment conducive to extensive tissue remodeling and fibrotic scarring, hallmark features of the severe clinical manifestations observed in GO.

TGF-β plays a direct and critical role in modulating secretion of various ECM proteins, including hyaluronan (HA), collagen, fibronectin, and proteoglycans, forming the structural scaffolding essential for tissue architecture, which are integral to scar tissue formation during the wound healing process. Specifically, the production of HA is a major contributor to the connective tissue swelling observed in GO, which is synthesized at the cell surface by the membrane-bound enzyme HA synthase (HAS). The HAS family, comprising HAS1, HAS2, and HAS3, is upregulated in response to several fibroblast-activating factors such as TGF-β, interleukin-1 (IL-1), insulin-like growth factor-1 (IGF-1), platelet-derived growth factor (PDGF), and leukoregulin [17,18]. This synthesis activity, enhanced by TGF-β among other cytokines, leads to HA accumulation, exacerbating the orbital tissue inflammation and expansion characteristic of GO.

TGF-β-induced synthesis of hyaluronan is mediated by PKC (protein kinase C) pathway activation in orbital fibroblasts. PKCs are a family of serine/threonine kinases, which often regulate cell growth and differentiation. The molecular structure of PKCs includes a highly conserved catalytic domain and a variable regulatory region, while the regulatory region of conventional PKCs contains a Ca^2+^-binding domain. 

TGF-β activates PKCβII, a key enzyme in HA synthesis regulation, leading to its translocation from cytoplasm to the cell membrane. In addition, TGF-β significantly induces mRNA expression of HAS2 and HAS3, followed by protein synthesis. The PKC inhibitor calphostin C, reduces HA synthesis induced by TGF-β. Moreover, the PKC activator PMA (phorbol 12-myristate 13acetate) stimulates HA synthesis in orbital fibroblasts [19]. On the other hand, PKA (protein kinase A) inhibits HA synthesis. 

Both PKA and PKC play contrasting roles in the regulation of HA synthesis in GO. Specifically, PKA acts as an inhibitor, while PKC serves as a stimulator of HA production. Notably, crosstalk between the PKA and PKC pathways is evident, with each influencing the activity of the other. The PKA inhibitor H-89 was found to stimulate HA synthesis in fibroblasts from GO patients. Increased intracellular free Ca^2+^ concentration was involved in TGF-β-stimulated HA synthesis, and Ca^2+^ chelator pre-treatment was proved to completely block the TGF-β-stimulated HA synthesis, indicating that TGF-β acts through a Ca^2+^-sensitive PKC to induce HA synthesis in orbital fibroblasts [17]. 

Connective tissue growth factor (CTGF) is a cysteine-rich, matricellular protein that is a key mediator in fibrotic diseases of organs such as the lung, liver, and kidney. The interaction between CTGF and TGF-β is significant in the various fibrotic processes. CTGF also participates in the TGF-β signaling pathway in the orbital fibrotic process, promoting myofibroblast differentiation and ECM production. Increased expression of fibrotic markers, including αSMA, CTGF, fibronectin, and collagen, has been observed in Graves’ orbital fibroblasts following stimulation by TGF-β1 [8]. In addition, oxidative stress significantly promotes the expression of CTGF, which can be attenuated by pre-treatment with antioxidants [8,20,21]. Moreover, the elevated levels of CTGF are associated with more severe clinical symptoms, including proptosis and eyelid edema [22]. These findings suggest that CTGF acts as an important downstream mediator of TGF-β-induced tissue remodeling in GO.

The accumulation of excessive ECM results from an imbalance between ECM production and degradation. Matrix metalloproteinases (MMPs) are important in degradation of ECM proteins, while tissue inhibitors of metalloproteinases (TIMPs) are endogenous inhibitors of MMPs. TGF-β regulates the expression of both MMPs and TIMPs, primarily promoting expression of TIMP-1 and TIMP-3 and decreasing expression of MMP-2/-9, thereby influencing the fibrosis process and ECM deposition in GO [23]. Kapelko-Słowik, K. et al. also showed that Graves’ disease patients exhibited higher serum concentrations of MMP-2 and MMP-9, as well as their inhibitors TIMP-1 and TIMP-2 [24]. Furthermore, they identified a positive correlation between elevated serum MMP-9 concentration and the clinical disease severity of GO and serum level of TSHR-Ab. TGF-β mediates ECM protein expression and tissue remodeling in the pathogenesis of GO, suggesting potential therapeutic targets for its treatment [25]. 

## 6. TGF-β Signal Pathways: Canonical and Non-Canonical Signaling Pathways

TGF-β exerts its multifaceted effects on fibrosis and inflammation through both canonical and non-canonical signaling pathways. 

### 6.1. Canonical Pathway

The canonical signaling pathway involves the activation of Smad proteins, which transmit signals from the cell surface to the nucleus, regulating gene expression associated with fibrosis and inflammation. 

Upon release from the cleavage of latency-associated peptide (LAP)–latent TGF-β-binding protein (LTBP) complex by matrix metalloproteinases (MMPs), the active TGF-β binds to TGF-β receptor 2 (TGFR2), initiating downstream signaling by activating TGF-β receptor 1 (TGFR1). Subsequently, activated TGFR1 phosphorylates Smad2 and Smad3, enabling their complex formation with Smad4, and begins nuclear translocation, modulating the transcriptional process of target genes that are essential for inflammation, proliferation, and remodeling [9]. 

Inside the nucleus, Smad3 directly interacts with gene promoters, inducing the transcription of profibrotic molecules including α-smooth muscle actin (α-SMA), collagen I, and tissue inhibitor of matrix metalloproteinases (TIMPs), facilitating myofibroblast activation and ECM production [26,27]. Moreover, Smad3 regulates the expression of profibrotic microRNAs (miRNAs) and long noncoding RNAs (lncRNAs), while also influencing epigenetic modifications to promote fibrosis. 

Bone morphogenetic protein-7 (BMP-7) is also an essential component of the TGF-β family. BMP-7 binds to BMP receptors, leading to the phosphorylation of Smad1, -5, and -8, which then form heteromeric complexes with Smad4. This complex translocate into the nucleus, where it exerts anti-fibrotic effects by inhibiting Smad3-dependent gene transcription. This process promotes the expression of anti-fibrotic molecules, contributing to the anti-fibrosis mechanism [28].

In contrast, Smad6 and Smad7 exert inhibitory effects on the Smad pathways. Smad6 blocks BMP-7 signaling by preventing Smad4 from binding to the phosphorylated Smad1, -5, and -8 complexes in the BMP-7 pathway [29]. Similarly, Smad7 acts as a negative regulator of Smad2/3 by recruiting Smurfs (Smad ubiquitin regulatory factors), which promote TGFR1 degradation and compete with Smad2/3 for binding with TGFR1. These exert inhibitory effects on fibrotic processes [30]. 

### 6.2. Non-Canonical Signaling Pathways

TGF-β can also activate non-canonical signaling pathways, including the mitogen-activated protein kinase (MAPK) pathways, phosphoinositide 3-kinase (PI3K) pathways, and RhoA-GTPase pathways, which regulate gene expression and contribute to cellular proliferation, differentiation, migration, and survival.

(1) MAPK Pathways

The TGF-β signaling pathways interact with the mitogen-activated protein kinase (MAPK) family, consisting of p38, c-Jun terminal kinase (JNK), and extracellular signal-regulated kinase (ERK), promoting orbital fibrosis via activating orbital myofibroblasts and overproducing ECM [23]. These kinases also interact with Smad signaling pathways, modulating gene expression and influencing cell growth, fibrosis, and inflammation.

TGF-β induces Ras activation, leading to sequential MEK1/2 and ERK1/2 activation. Activated ERK1/2 positively regulates Smad2 by increasing the amount of Smad2 protein and leading to enhanced transcriptional activity [31].

Additionally, TGF-β binding triggers TRAF4/6-mediated ubiquitylation and TAK1 activation, leading to p38/JNK activation. Activated p38/JNK enhances TGF-β signaling by phosphorylating Smad2 and Smad3 and other profibrotic transcription factors, like c-Jun and AP-1 [30]. 

In addition, upon analyzing the activities of tissue inhibitors of metalloproteinases (TIMPs) and matrix metalloproteinases (MMPs), which are involved in ECM metabolism downstream of the MAPK pathway, it was observed that TGF-β upregulates the expression of CTGF, α-SMA, and fibronectin, as well as TIMP-1 and TIMP-3, in human GO orbital fibroblasts, implying that the suppression of either the p38 or JNK pathway may provide a therapeutic potential to correct abnormal orbital tissue remodeling and fibrosis in GO [23].

(2) PI3K/Akt Pathways

Moreover, TGF-β can activate the phosphatidylinositol 3-kinase (PI3K)/Akt pathway, through activating Akt, and further inducing mTOR activation. This pathway promotes cell proliferation and migration and inhibition of fibroblast apoptosis in response to TGF-β signaling [32]. 

(3) Rho GTPase Pathway

TGF-β can also activate Rho GTPases, including RhoA (Ras homolog family member A), Rac1, and Cdc42, which regulate cytoskeletal remodeling, cell adhesion, and fibrosis. RhoA is a small GTPase that regulates actin cytoskeletal dynamics and cell contractility, while ROCK (Rho-associated protein kinase) is a downstream effector of RhoA that mediates cell migration, proliferation, and ECM deposition [33]. ROCK has two isoforms, ROCK1 and ROCK2. ROCK2 plays a key role in diabetic glomerulosclerosis and an ROCK2 inhibitor was observed to attenuate glomerular fibrosis in diabetic kidney disease [34]. 

In the pathogenesis of orbital tissue fibrosis in GO, aberrant activation of the RhoA/ROCK pathway contributes to the differentiation of fibroblasts into myofibroblasts and the subsequent deposition of ECM proteins [20], while the inhibition of the RhoA/ROCK pathway reduces myofibroblast differentiation and decreases the expression of profibrotic mediators, including α-SMA, CTGF, and collagen, induced by TGF-β. 

(4) Wnt/β-catenin Pathway

The Wnt/β-catenin pathway plays a crucial role in tissue repair, wound healing, and fibrosis. Activation of this pathway occurs when Wnt ligands bind to the Frizzled (FZD) receptor and LRP5/6 receptor, resulting in dephosphorylation, stabilization, and increased levels of β-catenin in the cytoplasm. Subsequently, β-catenin translocates into the nucleus and activates target gene expression, with these target genes being responsible for tissue repair, ECM deposition, fibrosis, and scarring [35]. A previous study has shown that the expression of Wnt and β-catenin is upregulated by TGF-β [36]. The crosstalk between TGF-β signaling and the Wnt/β-catenin pathway is mainly mediated by Smad proteins. Activation of the Wnt/β-catenin pathway induced by TGF-β and Smad2 phosphorylation results in increased expression of α-SMA and its incorporation into the stress fibers [37]. Additionally, Smad7 exerts an inhibitory effect on the activation of the Wnt/β-catenin pathway [38].

(5) NOX4/ROS pathway

The NOX4/ROS pathway is associated with excessive reactive oxygen species (ROS) production and serves as an important regulator in fibrosis, tissue remodeling, and inflammation. NADPH oxidases (NOXs) are enzymes that produce ROS, and NOX4 is an isoform of NOXs. Overexpression of NOX4 is linked to increased ROS accumulation, which exacerbates inflammation and remodeling by activating the NF-kB signaling pathway. Treatment strategies targeting the NOX4/ROS pathway have shown encouraging in vitro results in fibrotic diseases, such as cardiac remodeling [39,40]. Additionally, Oh et al. reported that TGF-β1 induces the production of NOX4 and excessive ROS, leading to mitochondrial damage. Treatment with an NOX4 inhibitor reduces ROS levels and TGF-β1-activated epithelial-to-mesenchymal transition, ultimately slowing down the peritoneal fibrosis process [41]. Furthermore, Xuan et al. revealed that downregulating NOX4 results in deactivation of the TGF-β1–Smad2/3 pathway and decreased expression of Smad2/3, leading to alleviation of airway inflammation and remodeling [42]. 

(6) Notch Pathway

The Notch pathway is involved in fibrosis and can be upregulated by TGF-β. TGF-β can interact with the Notch signaling pathway to regulate cell fate decisions, differentiation, and homeostasis. Additionally, TGF-β inhibitors downregulate the protein expression of Notch1 and Hes1, which are responsible for cell proliferation [43]. 

These non-Smad pathways provide additional layers of complexity and regulation to the TGF-β signaling network, allowing for diverse cellular responses to TGF-β stimulation. The integration of Smad and non-Smad signaling pathways is essential for the proper execution of TGF-β-mediated fibrosis, inflammation, and tissue remodeling, while the dysregulation of both pathways may result in pathological processes. The involved molecules represent a potential therapeutic strategy for attenuating TGF-β-induced fibrosis and inflammation.

## 7. TGF-β in Thyroid Homeostasis

Regulation of thyroid gland hemostasis involves complex interactions between thyroid-stimulating hormone (TSH), TGF-β, epidermal growth factors (EGFs), other growth factors, and cytokines. TGF-β serves as an important negative regulator of thyrocytes, inhibiting the proliferation and limiting the function of thyroid follicular cells in order to control thyroid gland homeostasis [44].

TGF-β signaling plays a crucial role in mediating immunity and inflammation in the thyroid, acting as a regulatory cytokine in prevention of autoimmune thyroid disease. It promotes the differentiation of regulatory T cells (Tregs) upon T cell receptor (TCR) activation, and inhibits the activation of effector T cells, thereby maintaining immune homeostasis and preventing excessive inflammation. TGF-β induces Th17 cell differentiation with IL-6 through activating Smad3 and STAT3, followed by the induction of the transcription factor ROR-γ [45]. Dysregulated TGF-β signaling can lead to aberrant immune responses, contributing to chronic inflammation in Graves’ ophthalmopathy [46].

## 8. Potential Therapeutic Targeting of TGF-β Pathways in GO

Since TGF-β plays a crucial role in the pathogenesis of GO, including tissue fibrosis, ECM accumulation, and orbital remodeling, various therapeutic strategies have been investigated to target TGF-β signaling pathways in GO treatment. Here, we summarize valuable insights from recent in vitro studies on the molecular and cellular mechanisms targeting TGF-β signaling in the fibrotic or inflammatory processes in GO eyes. We also discuss the therapeutic potential and challenges identified in clinical studies and trials focusing on GO. 

1. Sry-box transcription factor 9 (SOX9)

SOX9 is crucial for organ development, but its overexpression can promote fibrosis promotion by increasing gene expression responsible for excessive ECM accumulation. SOX9 is involved in the MAPK pathway and ERK1/2 pathways, contributing orbital fibrosis, upregulated by TGF-β [47,48]. Zhou et al. demonstrated that SOX9 is more highly expressed in orbital fibroblasts from eyes with GO compared to healthy controls, emphasizing its role as a positive regulator in differentiation and proliferation of these orbital fibroblasts and ECM remodeling [49]. They also reported that SOX9 knockdown significantly reduces the contraction and anti-apoptotic capacity of orbital fibroblasts and suppresses phosphorylated ERK1/2 expression. Additionally, SOX9 can bind to the EGFR promoter, inducing downstream fibrosis through a co-regulatory circuitry with EGFR. Thus, targeting SOX9 knockdown may be a therapeutic strategy for orbital fibrosis by inhibiting orbital fibroblast activation through the MAPK/ERK pathways induced by TGF-β.

To conclude, SOX9 is a critical factor in the pathogenesis of orbital fibrosis in GO, and targeting its pathways may offer a promising approach for therapeutic intervention.

2. Inhibition of RhoA and ROCK pathways: statins and ROCK inhibitors

RhoA and ROCK inhibitors, part of the downstream pathways of TGF-β signaling, may offer a potential therapeutic strategy for mitigating TGF-β-induced fibrosis and inflammation. Statins (3-Hydroxy-3-methylglutaryl-coenzyme A (HMG-CoA) reductase inhibitors), commonly prescribed for lowering low-density lipoprotein (LDL) cholesterol, also benefit fibrotic diseases like idiopathic pulmonary fibrosis and possess anti-inflammatory properties in cardiovascular diseases such as atherosclerosis [50,51]. Moreover, statins inhibit the post-translational modification of Rho family proteins, subsequently inhibiting the RhoA/ROCK pathway. This includes reducing the activity of MMP-2 and MMP-9 in ECM remodeling, which is upregulated by TGF-β [52]. 

Simvastatin, a member of the statin family, suppresses Ras family proteins in the RhoA, ERK, and MAPK pathways, thus inhibiting TGF-β-stimulated myofibroblast differentiation. Wei et al. showed that both simvastatin and the ROCK inhibitor Y-27632 cause a dose-dependent reduction of α-SMA expression in TGF-β-induced Graves’ orbital fibroblasts, achieved by blocking the RhoA/ROCK pathway and inhibiting ERK and p38 phosphorylation. In addition to the promising in vitro results demonstrating the anti-fibrotic effects of statins and ROCK inhibitors, clinical studies have observed protective effects against orbital fibrosis.

Elevated serum cholesterol levels are known to increase oxidative stress and proinflammatory cytokines secretions, which can enhance orbital fibroblast proliferation and IGF-1 secretion. A longitudinal cohort study showed that statin use in GO patients is associated with a decreased risk of developing thyroid-associated ophthalmopathy [53]. Naselii et al. reported that hypercholesterolemia is associated with a poorer response to intravenous steroid therapy in active GO patients, highlighting the importance of cholesterol-lowering treatments in GO patients, including statins [54]. Nilson et al. also noted that statins, rather than other lipid-lowering agents, had a protective effect against GO development in newly diagnosed Graves’ disease patients [55]. Moreover, a phase 2 randomized clinical trial (STAGO) demonstrated that adding statins to intravenous steroid therapy improved clinical outcomes in hypercholesterolemic patients with active moderate-to-severe GO [56]. However, further research is needed to evaluate the efficacy of statins in active GO patients regardless of their serum cholesterol level. A recent systematic review also revealed that statin therapy could prevent, maintain stability, and improve clinical symptoms with few adverse effects [57]. To summarize, while current research suggests that inhibiting the RhoA and ROCK pathways may benefit active GO patients, further preventive and therapeutic randomized trials are necessary to confirm these findings.

3. Suppression of CTGF

Connective tissue growth factor (CTGF) plays a key role in TGF-β-induced ECM production and myofibroblast transdifferentiation in orbital fibrosis in GO, with its level correlated with clinical severity. Knocking down CTGF expression in orbital fibroblasts using small hairpin RNA targeting the CTGF gene (shCTGF) significantly reduced TGF-β-induced expression of CTGF, along with fibronectin and α-SMA proteins, in Graves’ orbital fibroblasts. In addition, pre-treatment with antioxidants such as acetylcysteine and vitamin C has shown protective effects against oxidative stress-induced activation of CTGF [21]. 

Treatment strategies targeting CTGF are also being researched in idiopathic pulmonary fibrosis. Pamrevlumab, a fully human monoclonal antibody against CTGF, was reported to effectively slow disease progression safely in a phase 2, double-blind, randomized clinical trial (PRAISE study) [58]. However, the phase 3 randomized clinical trial (ZEPHYRUS-1 study) did not show a statistically significant improvement in the primary outcome, forced vital capacity, when treated with Pamrevlumab [59]. 

Despite inconsistent results in clinical trials for idiopathic pulmonary fibrosis, CTGF remains a central player in fibrosis and could still serve as a potential therapeutic target for orbital fibrosis in GO patients. Further exploration of CTGF suppression as a treatment strategy in GO is warranted. 

4. JNK and p38 inhibitors in MAPK pathway

In human Graves’ orbital fibroblasts, the MAPK pathway, including p38 and JNK signaling, mediates TGF-β1-induced myofibroblast activation and ECM overproduction. Hou et al. demonstrated that both p38 inhibitor (SB202190) and JNK inhibitor (SP600125) reduce the phosphorylation of p38 and JNK induced by TGF-β, and decrease the expression of fibrotic markers like α-SMA and fibronectin in response to TGF-β stimulation. Additionally, TIMPs, which are crucial in maintaining the balance of ECM deposition and degradation by inhibiting ECM degradation proteinases, are overexpressed (TIMP-1 and TIMP-3) in response to TGF-β, but their overexpression can be blocked by p38 and JNK inhibitors [23]. 

Given the potential of p38 and JNK inhibitors to prevent the fibrotic process in GO, further research is needed to explore this therapeutic target.

5. Curcumin

Curcumin (diferuloylmethane), a major constituent of turmeric (Curcuma longa), possesses numerous pharmacological activities, including anti-inflammatory, antioxidant, and antimicrobial properties [60]. It has been beneficial in various ophthalmological diseases, such as chronic anterior uveitis, dry eye syndrome, diabetic retinopathy, and age-related macular degeneration [61]. Curcumin’s anti-inflammatory effects include downregulating inflammatory cytokines such as cyclooxygenase-2 gene (COX-2), prostaglandin E2 (PGE-2), interleukins-1, -6, and -8 (IL-1, IL-6, IL-8), MMP-9, and tumor necrosis factor-α (TNF-α) by inhibiting the expression of nuclear growth factor κB (NF-κB). In addition, curcumin acts as a PPAR-γ agonist, inhibiting MAPK signaling pathways, reducing inflammatory markers, and decreasing oxidative stress [62]. Yu et al. demonstrated that curcumin suppresses the TGF-β1 signaling pathway and attenuates TGF-β1-induced myofibroblast differentiation in orbital fibroblasts, leading to a reduced expression of CTGF and α-SMA induced by TGF-β1 [63]. Despite curcumin’s limitations in terms of poor bioavailability, many studies have been conducted to increase its absorption efficiency [64]. 

In conclusion, curcumin shows promise as a potential therapeutic agent for the treatment of GO, warranting further research and development.

6. MicroRNA-29

Micro-ribonucleic acids (miRNAs) are endogenous, small, noncoding RNA molecules that regulate gene expression by inhibiting translation and deactivating target mRNA in the post-transcriptional stage [65]. MicroRNA-29, a recently discovered member of the miRNA family, is involved in the fibrosis process via inhibiting various pathways involved in ECM protein expression. Previous research has indicated that miRNAs play a significant role in cardiac fibrosis and idiopathic pulmonary fibrosis. The function and expression of MicroRNA-29 are closely associated with TGF-β. It has been observed that MicroRNA-29 decreases collagen production in fibroblasts, while its down-regulation using anti-miRNAs enhances collagen expression [66,67]. Treatment with TGF-β1 reduces the expression of MicroRNA-29 in orbital fibroblasts. MicroRNA-29 has also been shown to suppress TGFβ1-induced proliferation of orbital fibroblasts and mediate TGFβ1-induced ECM synthesis through the Wnt/β-catenin pathway in human orbital fibroblasts. It has been found that the expression of Wnt and β-catenin is upregulated by TGF-β. In contrast, microRNA-29 reduces the gene expression of fibrogenic genes, such as collagen type I alpha 1 (COL1A1), which is responsible for ECM deposition, by inhibiting the Wnt/β-catenin pathway [36]. 

Given the critical role of MicroRNA-29 in orbital fibrosis and abnormal ECM accumulation, it may serve as a valuable therapeutic target for GO. Further exploration of its potential could lead to new treatments for the condition.

7. Pirfenidone

Pirfenidone, a derivative of pyridinone, possesses both anti-inflammatory and anti-fibrotic properties. It is recognized as an appropriate treatment for idiopathic pulmonary fibrosis, supported by the CAPACITY trials [68]. In patients with thyroid-associated ophthalmopathy, pirfenidone inhibits IL-1β-induced hyaluronic acid production in orbital fibroblasts by suppressing MAPK-mediated hyaluronic acid synthase expression, showing greater potency than dexamethasone [69]. Moreover, Wu et al. conducted in vitro studies with orbital fibroblasts from GO eyes, revealing that pirfenidone reduces phosphorylation of p38 and JNK, thereby inhibiting fibrogenesis and attenuating the expression of fibrotic proteins induced by TGF-β1, including CTGF, fibronectin, α-SMA, and collagen type I. In addition, pirfenidone decreases ECM accumulation by deactivating TGF-β1-induced TIMP-1 protein expression and reactivating MMP-2 and MMP-9 activities [70]. 

To sum up, pirfenidone shows promise as a potential anti-fibrotic treatment for GO by reducing TGF-β1-induced fibrosis and ECM production in orbital fibroblasts. Further research could solidify its role as a therapeutic option in this condition. 

8. Antioxidants

Elevated levels of intracellular reactive oxygen species (ROS), increased oxidative DNA damage, and lipid peroxidation in orbital fibroblasts are key factors in the pathogenesis of GO [71]. The hypersensitivity of orbital fibroblasts in GO in response to oxidative stress exacerbates these conditions [72]. This increased oxidative stress results in elevated levels of TGF-β, IL-1, and the superoxide anion, which is much more apparent in GO orbital fibroblasts compared to normal controls [73]. Apart from suppressing CTGF expression induced by oxidative stress [21], antioxidants also exhibit protective effects against the production of proinflammatory cytokines and proliferation of orbital fibroblasts in GO. In vitro research has shown that pre-treatment with antioxidants, such as vitamin C and N-acetylcysteine, reduces intracellular IL-1 and TGF-β1 levels induced by oxidative stress, providing a theoretical basis for the treatment of GO [73].

Additionally, a recent study indicates that melatonin, another antioxidant, can attenuate bladder fibrosis and decrease expression of α-SMA and collagen III by inhibiting the TGF-β1–Smad pathways. Given its anti-fibrotic potential and ability to reduce fibrosis markers, it is suggested that melatonin may also be effective in treating orbital fibrosis, although further clinical evidence is needed [74].

Vitamin D plays a crucial role in modulating the synthesis of cytokines, such as IL-1 and IL-6, suppressing regulatory T cell activity, and negatively regulating lipogenesis. Consequently, vitamin D deficiency is associated with increased inflammation and a higher risk of autoimmune thyroid diseases. Given these associations, we recommend avoiding vitamin D deficiency in patients with GO to potentially mitigate these risks [75].

Furthermore, encouraging results have been reported regarding the clinical benefits of antioxidants in treating mild or inactive GO, including allopurinol, nicotinamide, pentoxifylline, selenium, and Enalapril; however, more research is needed to explore the potential of other antioxidants in targeting TGF-β signaling pathways in GO [76,77,78,79,80,81].

To conclude, antioxidants show potential in mitigating oxidative stress and fibrosis in GO, and further clinical studies are warranted to confirm their efficacy and expand the therapeutic options.

9. Sphingosine-1-phosphate (S1P)

Sphingosine-1-phosphate (S1P) is involved in the fibrosis process in GO orbital fibroblasts and the differentiation of orbital adipocytes. Kim et al. reported that expression levels of Sphingosine-1-phosphate receptor were significantly higher in GO orbital fibroblasts compared to a normal group. They also found that blocking this receptor reduced ROS generation and suppressed lipid accumulation [82]. Ko et al. demonstrated that the mRNA expression levels of Sphingosine-1-phosphate receptor and S1P increased significantly in GO orbital fibroblasts after treatment with TGF-β and cigarette smoke extract. Moreover, treatment with Sphingosine-1-phosphate receptor blockers significantly decreased the expression of profibrotic markers induced by TGF-β, such as collagen Iα, fibronectin, and α-SMA, thereby slowing the fibrosis process and tissue remodeling [83].

In brief, targeting the Sphingosine-1-phosphate pathway offers a promising therapeutic approach to mitigate fibrosis and tissue remodeling in GO, warranting further investigation.

## 9. Future Directions: Clinical Translation and Challenges

While preclinical studies have demonstrated promising results of various therapeutic strategies targeting TGF-β signaling in GO, clinical translation remains challenging. Factors such as off-target effects, dosing regimens, and patient heterogeneity pose significant difficulties in the development of TGF-β-targeted therapies. Future large-scale clinical research efforts are needed to overcome these challenges and optimize therapeutic strategies for effectively managing orbital fibrosis in GO and improving clinical outcomes and quality of life.

In our opinion, statins, as RhoA and ROCK inhibitors, hold significant promise for treating moderate-to-severe GO patients. Numerous clinical studies have already demonstrated the efficacy and safety of statins in these patients, although the results of phase 3 randomized controlled trials are still pending. In addition, therapeutic strategies targeting CTGF, such as monoclonal antibodies, have shown promising results in vitro and have been tested in large randomized clinical trials for idiopathic pulmonary fibrosis. Despite the disappointing outcomes in phase 3 clinical trials, targeting CTGF should not be dismissed and remains a potentially effective therapeutic strategy that deserves further exploration in GO patients. Moreover, clinical trials of various antioxidants, including allopurinol, nicotinamide, pentoxifylline, selenium, and enalapril, have shown favorable results in mild or inactive GO. We assert that further research focusing on moderate-to-severe GO is not only necessary but will also provide substantial benefits to patients.

## 10. Conclusions

Graves’ ophthalmopathy (GO) poses significant challenges due to its potentially vision-threatening symptoms and cosmetic concerns, which greatly impact patients’ quality of life.

TGF-β is a key player in the development of GO, driving the proliferation of orbital fibroblasts and excessive extracellular matrix accumulation, leading to orbital fibrosis and tissue remodeling. It mediates fibrosis and inflammation through both canonical and non-canonical signaling pathways, including the MAPK, PI3K/Akt, Rho GTPase, and Wnt/β-catenin pathways. Current treatments, primarily systemic steroids, often come with side effects and limited effectiveness. However, recent research highlights potential therapeutic targets related to TGF-β, such as SOX9, RhoA and ROCK inhibitors, connective tissue growth factor, JNK and p38 inhibitors, curcumin, microRNA-29, pirfenidone, antioxidants, and S1P.

In summary, this review provides a comprehensive overview of TGF-β’s role in the pathophysiology of GO, including inflammation, fibrosis, and immune dysregulation. It also explores recent studies on anti-fibrotic therapeutic targets associated with TGF-β. While targeting TGF-β signaling pathways appears promising for managing orbital fibrosis in GO, further research is essential to fully understand these mechanisms and enhance therapeutic strategies for better clinical outcomes.

## Data Availability

Not applicable.
